# *In situ* fabrication of porous biochar reinforced W_18_O_49_ nanocomposite for methylene blue photodegradation

**DOI:** 10.1039/d2ra02280j

**Published:** 2022-05-18

**Authors:** Yi Li, Wenting Chen, Zhiwei Liu, Dehua Cao, Yu Chen, Kunyapat Thummavichai, Nannan Wang, Yanqiu Zhu

**Affiliations:** Guangxi Institute Fullerene Technology (GIFT), Key Laboratory of New Processing Technology for Nonferrous Metals and Materials, Ministry of Education, School of Resources, Environment and Materials, Guangxi University Nanning 530004 China wangnannan@gxu.edu.cn Y.zhu@exeter.ac.uk; College of Engineering, Mathematics and Physical Sciences, University of Exeter Exeter EX4 4QF UK

## Abstract

In this paper, a novel cow dung based activated carbon (CDAC) was successfully modified by W_18_O_49_ nanowires as a photocatalyst using KOH activation and a hydrothermal method. The activity of photocatalytic degradation of methylene blue (MB) under full-spectrum light illumination shows great improvement, and the degradation rate of MB could reach 98% after 240 min (67% for W_18_O_49_), with a final degradation rate of 98%. The porous structure with specific surface area of CDAC (∼479 m^2^ g^−1^) increases the adsorption of W_18_O_49_ reactants and also raises the concentration of reactants in the photocatalytic region. The high electrical conductivity and good electron storage capacity of CDAC allow the electrons excited in the conduction band (CB) of W_18_O_49_ to migrate smoothly into CDAC, which are the keys to enhancing the photocatalytic activity. Moreover, the photocatalytic mechanism was proposed. The results show that the CDAC/W_18_O_49_ nanowire composite can be used as an efficient photocatalyst for removal of MB dye from wastewater and indicate remarkable future potential in dye wastewater treatment technologies.

## Abstract 2

Schematic illustration of the proposed reaction mechanism based on the CDAC/W_18_O_49_ composite.

## Graphical abstract

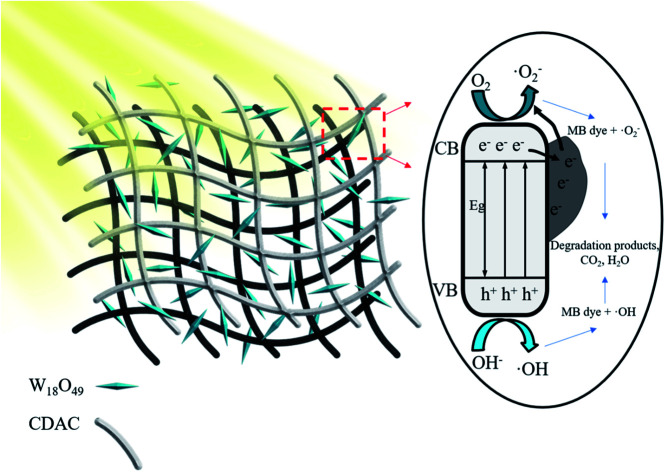

## 1. Introduction

With the rapid development of industrial processes, the problem of environmental pollution is receiving more and more attention. For a long time, many kinds of organic dyes have been widely used in various industries, such as tanneries, and paper and textile production,^1,2^ and the wastewater produced during their production is the main source of dye pollution in water bodies.^3–6^ Even low concentrations of dyes can cause great harm to humans, for example, methylene blue (MB) can cause serious skin problems, chromosome breakage, mutagenesis and human respiratory toxicity.^6–8^ Therefore, the challenge of how to remove organic dyes from wastewater has attracted widespread interest in the community.

To date, several techniques have been developed to address organic dyes in wastewater, such as solvent extraction, chemical oxidation, photocatalytic degradation, biodegradation, and adsorption.^9–15^ Among them, photocatalysis is a green, effective, environmentally friendly and highly promising advanced oxidation process for wastewater treatment, which uses semiconductor materials and light to remove organic pollutants. Tungsten oxide (WO_3−*x*,_ 0 ≤ *x* ≤ 1), a semiconductor material has the advantages of narrow band gap (range of 2.4 to 3.0 eV) favorable for visible light absorption, deep valence band for oxidation reaction, high carrier mobility and good stability.^16–19^ This makes WO_3−*x*_ a favorable candidate for solar-driven chemical reactions. Among them, non-stoichiometric W_18_O_49_ structure is rich in W^5+^ defects and oxygen vacancies, which can be used as reaction sites to facilitate the adsorption and activation of oxygen molecules, hence it has a significant important for the development of efficient photocatalysts for the removal of organic dyes from water.^20,21^

Unfortunately, the efficiency of narrow bandgap photocatalysts is relatively low due to the fast-compounding efficiency of photogenerated charge carriers, which inhibits the migration of these charges to reach the semiconductor surface to participate in redox reactions. In addition, their photostability is easily compromised because the oxidation and/or reduction potentials tend to lie within the band levels that induce photo-oxidation and/or reduction.^22^ For W_18_O_49_, it is easily deactive during photocatalysis due to the formation of WO_3_ by photooxidation of holes accumulated in the valence band.^21^ Therefore, the photocatalytic activity of most single-component photocatalysts is still far from satisfactory.^23^

In recent years, carbon-based-tungsten oxide composites have been studied more frequently to effectively improve the light-driven performance of W_18_O_49_ by taking advantage of the large specific surface area, flexible structure, excellent charge carrier mobility, and good electrical and thermal conductivity of carbon-based materials.^24,25^ Yang *et al.* reported a composite of highly ordered mesoporous WO_3_ nanocrystals grown on RGO, which was used as visible-light-driven photocatalyst for oxygen production. Under visible light irradiation, the amount of oxygen evolution from the optimized photocatalyst containing *ca.* 6 wt% RGO reached 437.3 μmol g^−1^, which was 5.1 times as high as that from m-WO_3_.^26^ Li *et al.* synthesized a lightweight 3-D porous aerogel using one-dimensional tungsten oxide nanowires and two-dimensional reduced graphene oxide sheets, and investigated the photocatalytic activity of the aerogel under visible light irradiation by degrading six different organic dyes.^18^ Deng *et al.* reported a rationally designed novel layered W_18_O_49_/g-C_3_N_4_ composite with enhanced photocatalytic activity by controlling the flow of dual-channel charge carrier separation and transfer processes, and the prepared composite exhibited enhanced photocatalytic performance under both full-spectrum light and near-infrared (NIR) light irradiation due to an effective strategy combining morphological structure and energy band structure modulation. Under optimal conditions, the degradation rate of W_18_O_49_/g-C_3_N_4_ composites to MB was 0.0677 min^−1^, which was 3 times and 5 times of g-C_3_N_4_(0.0276 min^−1^) and W_18_O_49_(0.0148 min^−1^), respectively; the removal of CIP by the W_18_O_49_/g-C_3_N_4_ composite reached 93.5% under full spectrum light (*λ* > 365 nm) irradiation for 120 min. For g-C_3_N_4_ and W_18_O_49_, 69.2% and 53.8% of CIP could be removed under the same conditions.^16^

Biochar is a cheap and green carbon-based material obtained by pyrolysis of biomass feedstock at high temperatures and under anaerobic conditions.^27^ Biochar has a high surface area and porous structure, structural defect sites and various surface functional groups, which provide excellent electrical conductivity and electron storage capacity in photocatalytic processes.^28^ Electrons that leap under light can be transferred to biochar, contributing to a lower electron–hole complexation rate in the photocatalytic process, which improves the oxidative removal of target compounds, while the raw material is renewable and easily available.^29,30^ Cow dung is a common livestock waste product, which is mainly derived from undigested cellulose-based feed residues and without proper treatment will cause environmental problems such as deterioration of air quality, public hazards (*e.g.*, infectious pathogens and asphyxiation poisoning), greenhouse gas emissions, and water pollution.^31^ Currently, cattle manure is used by some as cooking fuel, disinfectant cleaner, construction material, insulation material, waterproofing material for walls and floors of rural houses, and for electricity generation. There is also the use of cattle manure as a raw material for the preparation of biochar, which can be an effective solution for a large amount of livestock waste.^32,33^ Thus, cattle manure can be used as a cheap and abundant source of carbon material.

In this paper, high specific surface area porous structure cow dung active carbon (CDAC) was prepared for the first time by using cow dung as carbon raw material and active by KOH, and W_18_O_49_ was loaded on CDAC by hydrothermal method to synthesize CDAC/W_18_O_49_ composites, which benefited from the large specific surface area structure of CDAC and the interface between amorphous carbon CDAC and W_18_O_49_ could extend the current The photocatalytic degradation of MB by CDAC/W_18_O_49_ composites was enhanced by the large surface area structure of CDAC and the interface between amorphous carbon CDAC and W_18_O_49_, which could extend the current carrier lifetime and accelerate the charge transfer. In addition, a schematic diagram of the reaction mechanism was constructed, and a possible photocatalytic mechanism was proposed.

## 2. Experimental section

### 2.1. Materials

Cow dung was obtained from Guangxi Buffalo Research Institute (Nanning, China). Tungsten hexachloride (WCl_6_), cyclohexanol and methylene blue (MB) were purchased from Shanghai Macklin Biochemical Co. Potassium hydroxide (KOH), disodium EDTA-2Na, anhydrous ethanol, *tert*-butanol (*t*-BuOH), hydrochloric acid (HCl) and 1,4-benzoquinone (BQ) were purchased from Guandong Guanghua Sci-Tech Co. Deionized (Dl) water was obtained from an ultrapure water production facility and used throughout the experiments. All chemicals and reagents were used as received without further purification.

### 2.2. Preparation of the cow dung active carbon

CDAC was synthesized by chemical activation using KOH as the activator and pre-carbonized cow dung charcoal as the precursor. First, the sun-dried cow dung was pre-carbonized in the tube furnace at 450 °C for 2 h under an argon gas flow rate of 50 ml min^−1^. Then, the charcoal was mixed with KOH in a 1 : 2 ratio. The mixture was transferred to an alumina crucible and pyrolyzed at a rate of 10 °C min^−1^ in a constant stream of argon at 800 °C, and then held for 2 h before natural cooling. The obtained product was washed with 1.0 M hydrochloric acid to remove residual KOH, followed by DI water until the filtrate became neutral. Finally, the fabricated CDAC was collected after drying at 80 °C overnight.^32^

### 2.3. Preparation of the CDAC/W_18_O_49_ composite

Firstly, 40 mg of CDAC obtained was added to 70 ml of cyclohexanol solution and stirred at 40 °C for 1 h to obtain a homogeneous mixture. Then, 83.3 mg of WCl_6_ was dissolved in 70 ml of the mixed solution and kept stirring for 15 min. Then mixture was sealed in an autoclave with a Teflon liner and heated at 200 °C for 6 h. Finally, the black-blue precipitate was collected by centrifugation, washed several times with ethanol and deionized water, and dried in vacuum at 60 °C for 10 h. For comparison, pure W_18_O_49_ was also synthesized in the same way, without the addition of CDAC.

### 2.4. Characterization of materials

The crystal structure of the catalysts was characterized with Cu Kα1 radiation (*λ* = 1.54056 Å) using an X-ray diffractometer (XRD, Rigaku D/MAX 2500 V, Rigaku Corporation) at an accelerating voltage of 45 kV, scan rate (2*θ*): 0.05° s^−1^, recording a 2*θ* range of 10° to 80°. Scanning electron microscopy (SEM, Sigma 300, Carl Zeiss) was used to study the microstructure and morphology of the prepared photocatalysts. The FT-IR of the synthesized photocatalysts was collected by transmission electron microscopy (TEM) and high-resolution transmission electron microscopy (HR-TEM) (F20 S-TWIN electron microscope, Tecnai G2, FEI Co.) at an accelerating voltage of 200 kV. FT-IR spectra of the synthesized photocatalysts were collected by Fourier transform infrared spectrometer (IRAffinity-1, Shimadzu, Japan). A surface area analyzer (TriStar II 3020, Micromeritics) was used to analyze the specific surface area and pore volume of the synthesized photocatalysts. To obtain Raman spectra, a microscopic Raman spectrometer (HORIBA Jobin Yvon, Lab RAM HR Evolution) with Raman shifts measured between 100 cm^−1^ and 2000 cm^−1^ was used. X-ray photoelectron spectroscopy (XPS) of the prepared samples was performed on an XPS spectrometer with Al Kα source (XPS, ESCALAB 250XI, Thermo Fisher), exploring the valence states of W and C elements in the prepared samples. The UV-vis-NIR diffuse reflectance spectra (UV-vis-NIR DRS) of the prepared samples were performed by an UV-vis-NIR spectrophotometer equipped with an integrating sphere (PerkinElmer LAMBDA 365 UV/Vis Spectrophotometer) using BaSO_4_ as a reference.

### 2.5. Photoelectrochemical measurement

A typical three-electrode measurement system based on the CHI 660D workstation was applied to measure the photoelectrochemical properties of the as-prepared samples. Pt electrode and an Ag/AgCl electrode in saturated KCl solution were used as counter electrode and reference electrode, respectively. The photocurrent density was measured in a 1 M sulfuric acid electrolyte solution under visible light provided by a 300 W Xe arc lamp (*λ* > 365 nm). Electrochemical impedance spectroscopy (EIS) was measured based on the photoelectrochemical test system described above.

### 2.6. Photocatalytic activity measurement

The photocatalytic activity of the prepared photocatalysts was investigated by degrading MB under light irradiation. A 300 W xenon lamp (PLS/SXE 300C, Perfectlight Co., Ltd, Beijing) was employed as the light source, and it was directly used as a full-spectrum light source without any filter. Before the photocatalytic experiment started, 10 mg of the prepared photocatalyst was added into 100 mL of 35 mg L^−1^ MB aqueous solution and stirred for 60 min to reach the adsorption equilibrium under protection from light. During the photocatalytic process, the quantitative aqueous solution was collected at certain time intervals, and the photocatalyst was removed by centrifugation and analyzed.

## 3. Results and discussions

### 3.1. Structure and morphology characteristics

**Fig. 1:**
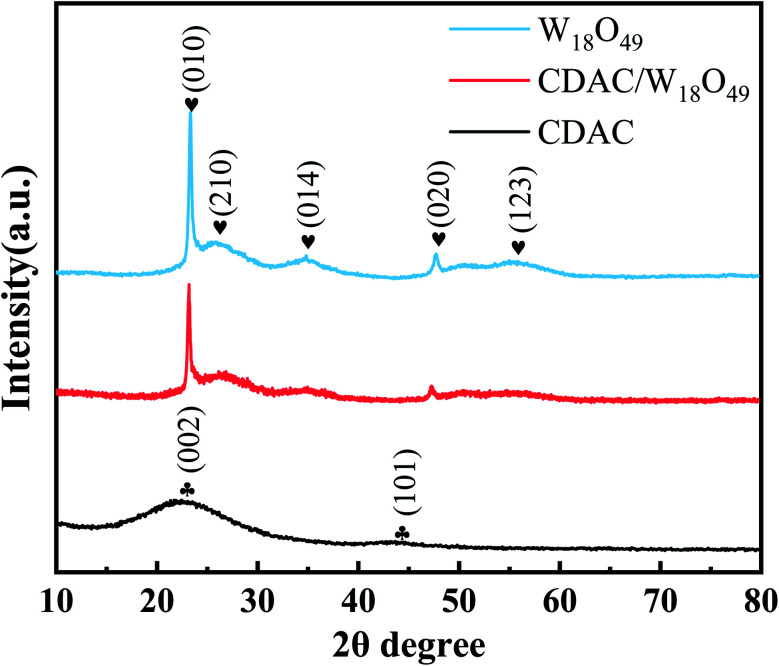
XRD patterns of the prepared CDAC, W_18_O_49_ and CDAC/W_18_O_49_.

Fig. 1 shows two diffraction peaks are observed at 25° and 43° for cow dung active carbon, corresponding to the graphitic phase structure on the (002) plane and the chaotic carbon layer structure on the (101) plane. The spectra of W_18_O_49_ nanowires show characteristic peaks at 23.4°,25.8°, 34.9°, 47.8° and 55.6° positions corresponding to (010), (210), (014), (020) and (123) crystallographic planes, respectively, which can be well matched with JCPDS No. 05-0392 (monoclinic W_18_O_49_) card, with (reported) The peak positions are consistent.^25^ Although most of the diffraction peaks of CDAC/W_18_O_49_ composites correspond to the characteristic peaks of the monoclinic system W_18_O_49_, it can also be seen that the characteristic peaks of CDAC/W_18_O_49_ composites are shifted to the lower 2*θ* side compared to W_18_O_49_, which may be due to the lattice expansion when W_18_O_49_ forms composites with CDAC further supporting the high concentration of oxygen in the nanocomposites high concentration of vacancies; meanwhile, it can be observed that the characteristic peaks of CDAC in CDAC/W_18_O_49_ composites are not obvious, which may be caused by the low CDAC content and the poor crystallinity of CDAC/W_18_O_49_ composites. In addition, no peaks of other impurities were detected in the XRD patterns of the CDAC/W_18_O_49_ composites.

**Fig. 2:**
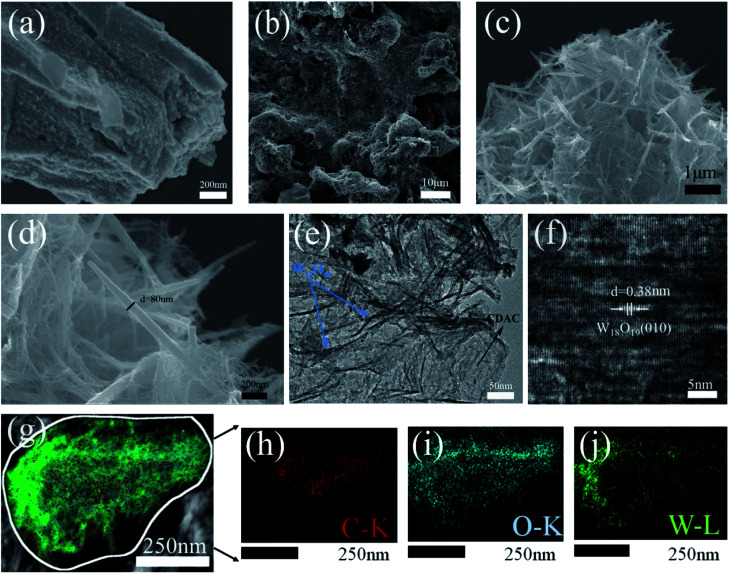
SEM images of (a) CDAC (b–d) CDAC/W18O49 (e and f) TEM image of CDAC/W18O49 (g) the corresponding elemental mapping images of CDAC/W18O49 composites and (h–j) corresponding EDS mapping image of C, O and W element.

The surface morphology of the catalysts was investigated by SEM and TEM. Fig. 2a shows SEM image of pristine CDAC shows a rough surface filled with porous morphology. After passing the hydrothermal reaction, the surfaces of CDAC are modified with linear W_18_O_49_ (as in Fig. 2b). Further observation by higher magnification SEM images (Fig. 2c and d) shows that the W_18_O_49_ nanowires on CDAC have diameters of 20–60 nm and lengths of 0.3–0.8 μm. Fig. 2e represents transmission electron microscopy (TEM) images of individual CDAC/W_18_O_49_ composites, which can be seen to be modified with uniformly sized. Fig. 2f shows elemental mapping image of the CDAC/W_18_O_49_ composite shows that the C elements are mainly distributed in the middle region, while the O and W elements are mainly distributed on the outer surface of the C elements. HRTEM image (Fig. 2g) shows that the lattice stripe spacing of W_18_O_49_ nanowires on the surface of CDAC is ∼0.38 nm, which is consistent with the lattice spacing of the (010) crystal plane of monoclinic W_18_O_49_, indicating that W_18_O_49_ grows along the (010) direction.

### 3.2. Specific surface area measurement

**Fig. 3:**
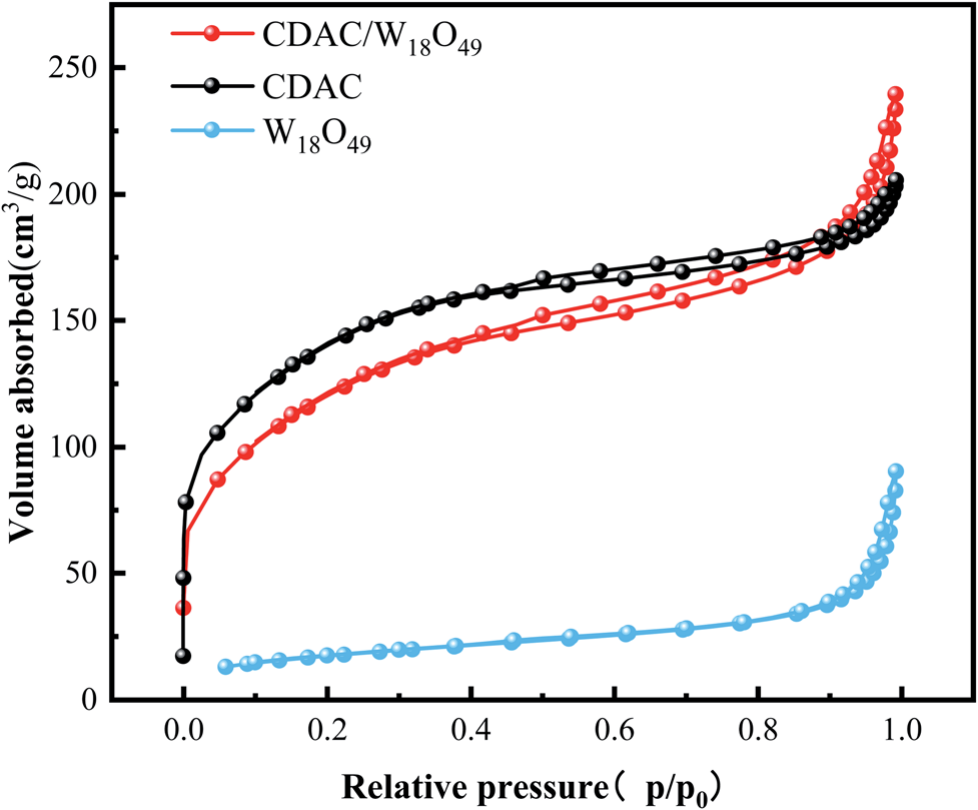
N_2_ adsorption–desorption isotherm of CDAC, W_18_O_49_, CDAC/W_18_O_49_ composites.

The pore structures and SSA of the materials CDAC, W_18_O_49_, and CDAC/W_18_O_49_ were analyzed by the adsorption and desorption of N_2_. The N_2_ adsorption and desorption curves of CDAC, W_18_O_49_, and CDAC/W_18_O_49_ composites are shown in Fig. 3. According to the classification criteria of the International Society for Pristine and Applied Chemistry, the curves can be classified as a combination of Class I and IV curves.^34^ At relative pressures *P*/*P*_0_ < 0.1, the CDAC and CDAC/W_18_O_49_ curves show a significant increase in N_2_ adsorption, indicating the presence of a large number of micropores in the material, and at relative pressures in the range of 0.41 < *P*/*P*_0_ < 0.95, there is a significant hysteresis loop in both curves, indicating the presence of mesopores in the material, but the adsorption and desorption curves of W_18_O_49_ are not significant. When the relative pressure was in the range of *P*/*P*_0_ > 0.95, the adsorption and desorption curves were close to vertical, suggesting the presence of macroporosity in all three materials. In addition, the SSA of CDAC is 479.1034 m^2^ g^−1^, which is larger than that of CDAC/W_18_O_49_, but the relative pore volume is smaller than that of CDAC/W_18_O_49_, as shown in Table 1, which may be caused by the larger number of mesopores in CDAC and the larger number of macropores in CDAC/W_18_O_49_, which is consistent with the adsorption–desorption curve. The larger pore volume is beneficial to promote the photocatalytic effect of CDAC/W_18_O_49_ on MB.

**Table 1:** Specific surface area and pore volume of the as-prepared samples

Samples	Specific surface area (SSA) (m^2^ g^−1^)	Pore volume (cm^3^ g^−1^)
CDAC/W_18_O_49_	422.1559	0.355
CDAC	479.1034	0.312
W_18_O_49_	59.2412	0.123

### 3.3. Surface chemical composition and group analysis

**Fig. 4:**
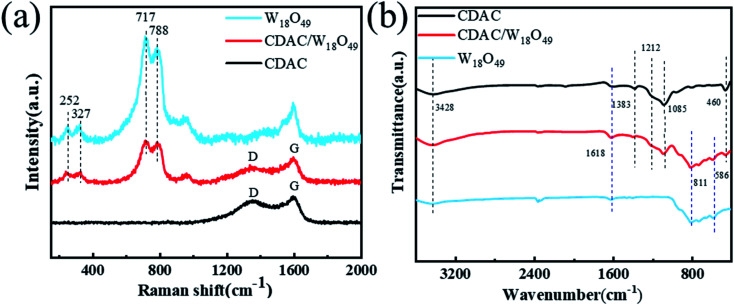
(a) Raman spectra of prepared CDAC, W_18_O_49_ and CDAC/W_18_O_49_; (b) FT-IR spectra of CDAC, W_18_O_49_ and CDAC/W_18_O_49_.

Fig. 4a shows the Raman spectra of CDAC/W_18_O_49_ composites compared with the Raman spectra of CDAC and W_18_O_49_ nanowires. CDAC has two broad peaks at 1351 and 1595 cm^−1^ corresponding to the hybridized carbon atoms assigned to sp^3^ (D-band) and sp^2^ (G-band), respectively.^35^ The Raman spectra of W_18_O_49_ nanowires is dominated by four major vibrational peaks at 252, 327, 717 and 788 cm ^−1^, attributed to the bending vibration of monoclinic phase W_18_O_49_*δ* (O–W–O) and the stretching vibration of *ν* (W–O–W), respectively.^36^ The Raman spectra of the CDAC/W_18_O_49_ composites show that they are comparable to the CDAC and W_18_O_49_ nanowires corresponding to the characteristic Raman peaks of the nanowires.

To further investigate the atomic structure of the CDAC/W_18_O_49_ composite, FT-IR tests were performed, as shown in Fig. 4d. There are five peaks in the spectrum of CDAC, the peak at 460 cm^−1^ is attributed to Si–O–Si bending vibration, the peak at 1085 cm^−1^ is attributed to C–C stretching vibration, the peaks at 1212 and 3428 cm ^−1^ are attributed to the –OH stretching mode, and the peak at 1383 cm ^−1^ is attributed to the medium C–O bond axial deformation vibration. –OH stretching mode, and the peak at 1383 cm^−1^ are an axial deformation vibration in the C–O bond. Characteristic stretching vibrational bands belonging to W=O and O–W–O (500–1000 cm^−1^) are observed in the FTIR spectra of the W_18_O_49_ nanowire. After introducing W_18_O_49_ nanowires into CDAC, their FTIR spectra reveal the characteristic peaks of W_18_O_49_ nanowires and CDAC.

**Fig. 5:**
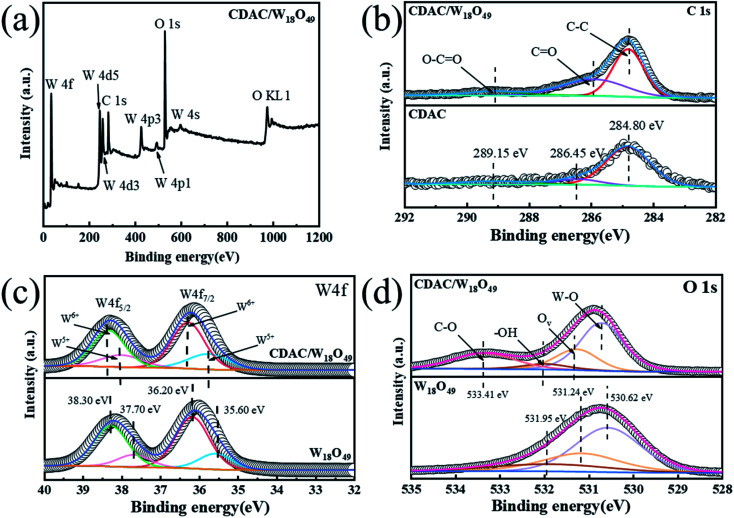
The XPS spectra of the prepared CDAC/W_18_O_49_, W_18_O_49_ and CDAC: (a) survey, (b) C 1s, (c) W 4f, (d) O 1s.

X-ray photoelectron spectra (XPS) spectra of the prepared samples are also provided to further investigate the interactions between CDAC and W_18_O_49_. The XPS spectra in Fig. 5a show that the prepared CDAC/W_18_O_49_ composites are mainly composed of C, W and O elements. Fig. 5b–d show the high-resolution spectra of C 1s, W 4f and O 1s. As shown in the high-resolution C 1s spectrum of CDAC in Fig. 5b, the peak at the binding energy of 284.80 eV is mainly attributed to C–C of surface amorphous carbon, while the peaks at 286.45 and 289.15 eV are attributed to C=O and O–C=O, respectively.^37^ These three peaks can also be observed on the curves of CDAC/W_18_O_49_ samples. However, unlike the pure CDAC, the characteristic peaks of C=O and O–C=O in CDAC/W_18_O_49_ are shifted to the side with lower binding energy after adhering to the W_18_O_49_ nanowire. In the W 4f high-resolution XPS spectrum of pure W_18_O_49_ (Fig. 4a), the main spectrum is divided into two pairs of peaks, which represent two different oxidation states of element W, namely W^6+^ and W^5+^. In the W 4f high-resolution XPS spectrum of pure W_18_O_49_ (Fig. 5c), the main spectrum is divided into two pairs of peaks, which represent two different oxidation states of element W, namely W^6+^ and W^5+^. In the W 4f high-resolution XPS spectrum of pure W_18_O_49_ (Fig. 5c), the main spectrum is divided into two pairs of peaks, which represent two different oxidation states of element W, namely W^6+^ and W^5+^. The peaks with binding energies of 36.20 and 38.30 eV can correspond to the W 4f_7/2_ and W 4f_5/2_ characteristic peaks of W^6+^, respectively. The second double peak, with binding energies of 35.60 and 37.70 eV, corresponds to the W 4f_7/2_ and W 4f_5/2_ characteristic peaks of W^5+^. In addition, it can be seen that the W 4f high-resolution XPS spectrum of CDAC/W_18_O_49_ exhibits a similar peak to the pure W_18_O_49_ spectrum with a slight offset. The position of the W 4f peak of CDAC/W_18_O_49_ is shifted toward higher binding energy values compared to pure W_18_O_49_, which can be attributed to the interaction between W_18_O_49_ and CDAC.^38^ In the O 1s high-resolution XPS spectrum of W_18_O_49_ (Fig. 5d), the peaks located at 530.62, 531.34 and 531.95 eV are attributed to W–O, oxygen vacancy (O_v_) and –OH, respectively, and the characteristic peaks of W–O, oxygen vacancy (O_v_) and –OH are shifted to the higher binding energy side after the composite formation with CDAC. Notably, a new peak appears at 533.41 eV on the O 1s spectrum of the CDAC/W18O49 sample, which may be related to the C–O bonding because of the close contact and reaction between the CDAC and W_18_O_49_ nanowires. The shift of the binding energy on the XPS curve indicates the electron migration between W_18_O_49_ and CDAC, due to their different electron concentrations, electrons are more willing to transfer from W_18_O_49_ nanowires to CDAC.^39^ Based on the above analysis results, it can be confident that the W_18_O_49_ nanowires have been successfully assembled onto the CDAC.

### 3.4. Optical properties

**Fig. 6:**
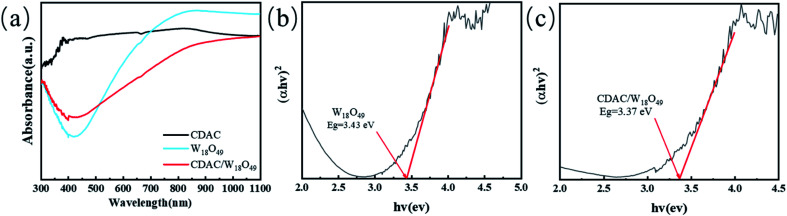
(a) UV-vis-NIR diffuse reflectance spectra of the prepared CDAC, W_18_O_49_, and CDAC/W_18_O_49_; (b and c) the band gap energy of W_18_O_49_ and CDAC/W_18_O_49_.

The light trapping ability of CDAC, pristine W_18_O_49_ nanowires and CDAC/W_18_O_49_ composites was investigated by UV-vis-NIR absorption spectroscopy, and the relevant results are shown in the Fig. 6a. For CDAC showed significant absorption within the full spectrum.^40^ The pristine W_18_O_49_ nanowires have a primary absorption edge of 440 nm and an absorption region ranging from 440 to 1100 nm, showing strong responsiveness throughout the visible region and near-infrared light range. This unique light absorption ability originates from the metal-like LSPR effect of the W_18_O_49_ nanowires due to the presence of abundant oxygen vacancies.^41,42^ When CDAC was compounded with W_18_O_49_ nanowires, it was seen that the CDAC/W_18_O_49_ composites exhibited significant absorbance and a main absorption edge consistent with that of pristine W_18_O_49_ nanowires, further confirming the presence of W_18_O_49_ nanowires.

To fully understand the variation in light absorption capacity, the band gaps (*E*_g_) of the W_18_O_49_ nanowires and CDAC/W_18_O_49_ composites were calculated by the following equation.^23^

1 *αhv* = *A*(*hv* − *E*_g_)^*n*/2^

where *α*, *h*, *v*, *E*_g_ and *A* are the absorption coefficient, Planck's constant optical frequency, band gap energy and constant, respectively. Based on the results shown Fig. 6b and c, the W_18_O_49_ nanowire and CDAC/W_18_O_49_ composite are estimated to be 3.43 and 3.37 eV, respectively.

### 3.5. Photogenerated charge transport properties and transfer properties

**Fig. 7:**
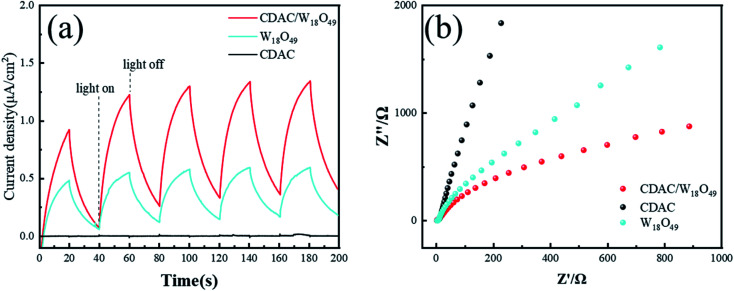
(a) Photocurrent response of W_18_O_49_ nanowires CDAC and CDAC/W_18_O_49_. (b) Electrochemical impedance spectroscopy (EIS) of W_18_O_49_ nanowires CDAC and CDAC/W_18_O_49_ composites.

To comprehensively investigate the photocurrent response and photogenerated charges compounding efficiency of the photocatalysts, transient photocurrent (It) and electrochemical impedance spectroscopy (EIS) were used to measure the charge transfer of the materials. The CDAC/W_18_O_49_ composite and W_18_O_49_ nanowires exhibited a significant photocurrent response during light irradiation in Fig. 7a. The CDAC/W_18_O_49_ composite exhibited a higher photocurrent density than that of the W_18_O_49_ nanowires, showing higher light trapping and lower photogenerated charge complexation efficiency, while the pristine CDAC showed no photocurrent response. EIS is an effective electrochemical approach to explain the electron transfer efficiency of the photocatalyst. Fig. 7b shows that the CDAC/W_18_O_49_ composite possesses the smallest diameter, which implies the lowest charge transfer impedance and complexation efficiency of photogenerated charges.

### 3.6. Photocatalytic activity of the prepared photocatalysts

**Fig. 8:**
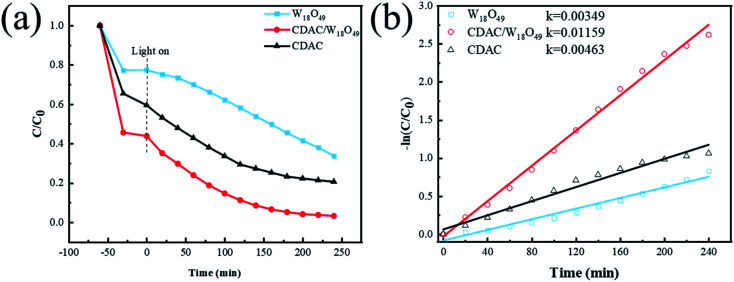
(a) Photocatalytic degradation of MB with different samples under full-spectra light irradiation (*λ* > 365 nm) and (b) the corresponding pseudo-first-order kinetic plots.

The photocatalytic performance of the prepared CDAC/W_18_O_49_ composites was investigated by the degradation of methylene blue (MB). Adsorption experiments were performed under dark conditions prior to the photocatalytic degradation process to investigate the adsorption capacity of different photocatalysts. The results showed that the adsorption–desorption equilibrium between the photocatalyst and the pollutant molecules could be achieved within 60 min. Fig. 8a shows the MB degradation results for pristine W_18_O_49_ nanowires, CDAC and CDAC/W_18_O_49_ composites. The results show that for pristine W_18_O_49_ nanowires, only 67% of MB was degraded after 240 min of full spectrum irradiation (*λ* > 365 nm), while for CDAC, only 80% of MB was removed. When the prepared CDAC/W_18_O_49_ composites were added to the reaction system, the photocatalytic removal efficiency was improved. In Fig. 8a, it can be observed that the CDAC/W_18_O_49_ composite has the highest photocatalytic activity and can degrade 90% of MB molecules at 120 min of light, and the removal rate can reach 98% after 240 min. The better photocatalytic activity of CDAC/W_18_O_49_ composites may be attributed to the good electrical conductivity of CDAC as an electron absorber and transport network, the increased specific surface area of the composites with CDAC as a substrate, which allows more exposed active sites, and the lower band gap of W_18_O_49_ nanowires and the defect structure caused by numerous oxygen vacancies.

In addition, the kinetics of photocatalytic degradation of MB under full-spectrum light irradiation was investigated, and the results showed that the variation of MB concentration *versus* reaction time on CDAC/W_18_O_49_ composites followed a pseudo-first-order kinetic diagram with the equation – ln(*C*/*C*_0_) = *kt*, where *t*, *C*_0_ and *C* are the reaction time, initial methyl concentration (mg L^−1^), and methyl at time *t*, respectively concentration (mg L^−1^). *k* represents the apparent pseudo primary rate constant (min^−1^). The pseudo primary rate constants of pristine W_18_O_49_ nanowires, CDAC/W_18_O_49_ composites and CDAC are 0.00349, 0.01159 and 0.00463 min^−1^, respectively, as can be seen in Fig. 8b. The pseudo primary rate constants of CDAC/W_18_O_49_ composites are higher than those of pristine W_18_O_49_ nanowires and CDAC, respectively. W_18_O_49_ nanowires and CDAC by a factor of about 3.3 and 2.5, respectively.

**Fig. 9:**
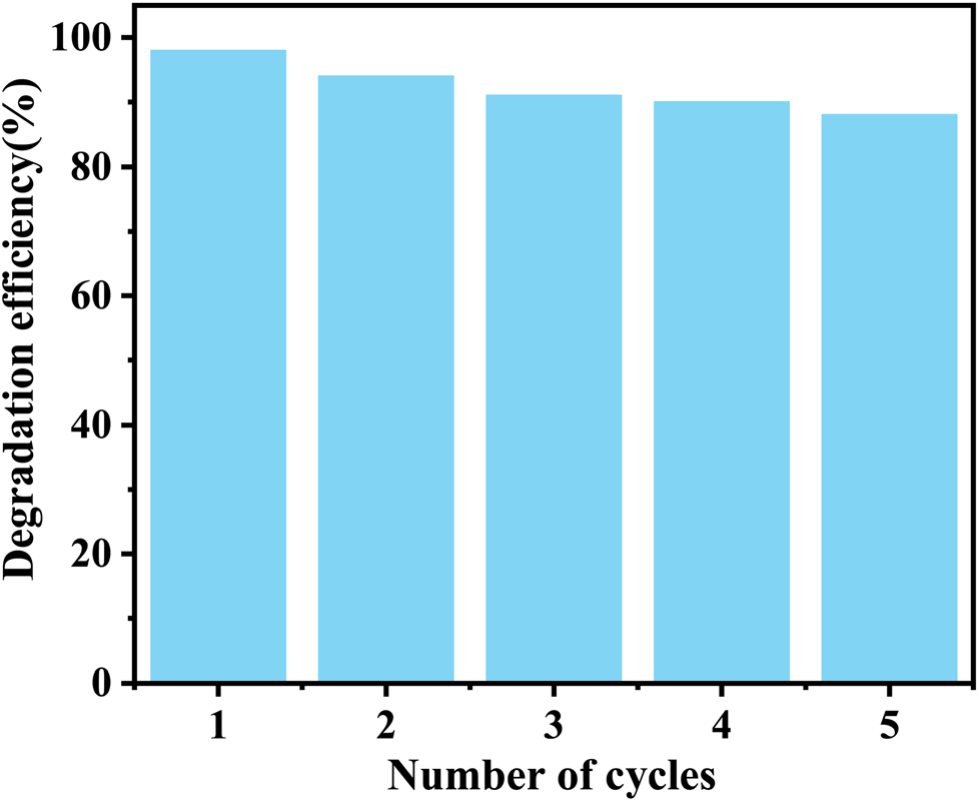
Recyclability tests of CDAC/W_18_O_49_ composites in degradation of MB dye.

For practical applications, the reusability and stability of photocatalysts are very important, therefore, to evaluate the photostability of CDAC/W_18_O_49_ composites, cycling reactions were performed. After each catalytic run, the photocatalyst was separated from the solution, washed with ethanol and vacuum dried to ensure the purity of the recovered catalyst. As shown in Fig. 9, after five cycling experiments, the photocatalytic activity of CDAC/W_18_O_49_ did not show a significant degradation process, indicating that the prepared CDAC/W_18_O_49_ composites have high stability.

### 3.7. Photocatalytic reaction mechanism

**Fig. 10:**
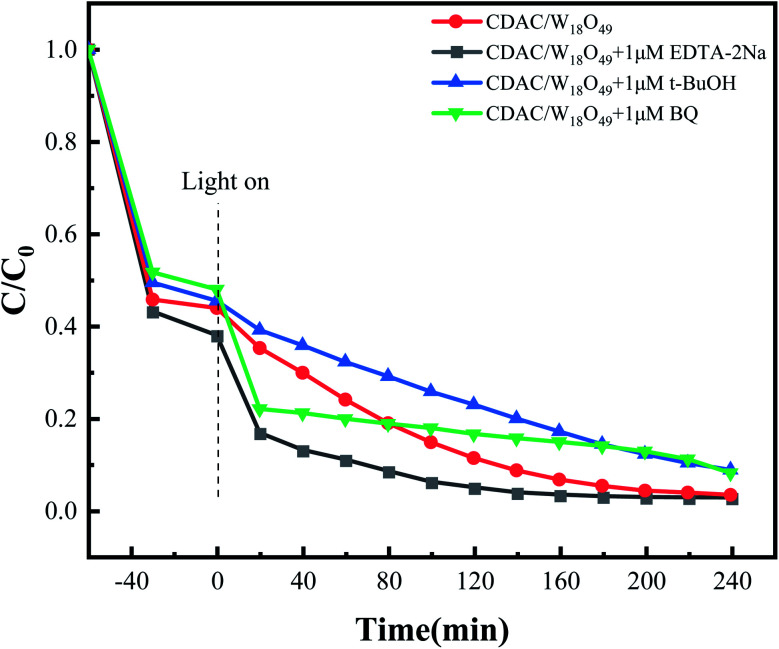
Trapping experiments for the photocatalytic degradation of MB under full-spectra light irradiation (*λ* > 365 nm).

In order to understand the photocatalytic mechanism of this composite, controlled experiments were performed to capture radicals, with EDTA-2Na, *t*-BuOH and BQ as hole (h^+^), hydroxyl radical (˙OH) and superoxide radical (˙O_2_^−^) trapping agents, respectively. As shown in the Fig. 10, when 1 μM 1,4-benzoquinone (BQ) was added to the reaction system, the photocatalytic degradation efficiency of MB under full-spectrum light irradiation (*λ* > 365 nm) was somewhat suppressed, indicating that superoxide radicals (˙O_2_^−^) play some roles in the photocatalytic degradation process. The photocatalytic activity of CDAC/W_18_O_49_ composites was significantly reduced when 1 μM *tert*-butanol (*t*-BuOH) was added, which indicated that hydroxyl radicals (–OH) played a major role in the photocatalytic degradation process. In contrast, the degradation rate of MB was significantly increased by adding 1 μM disodium ethylenediaminetetraacetate (EDTA-2Na). The reason why EDTA-2Na can improve the degradation rate is that it can trap the hole (h^+^), so that more ˙O_2_^−^ and ˙OH reactive substances in the system can participate in the reaction, thus improving the degradation rate of MB.

**Fig. 11:**
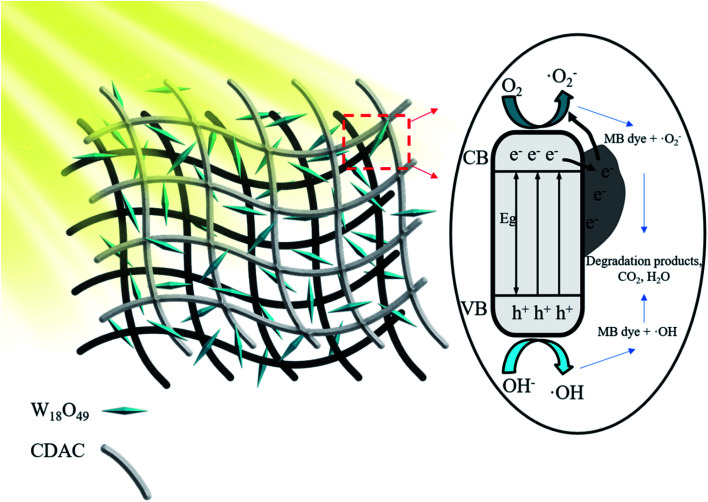
Schematic illustration of the proposed reaction mechanism based on CDAC/W_18_O_49_ composite under full-spectrum light (*λ* > 365 nm).

In summary, a possible mechanism for the photocatalytic degradation of methylene blue (MB) by CDAC/W_18_O_49_ composite is proposed. As shown in Fig. 11 under full-spectrum light irradiation, W_18_O_49_ can absorb enough energy to generate excited electrons and form electron–hole pairs. The excited electrons in the conduction band (CB) of W_18_O_49_ can migrate smoothly into the CDAC due to the high conductivity and good electron storage capacity of CDAC, as evidenced by the photocurrent response and EIS. Thus, the combination of photogenerated carriers can be effectively suppressed and the absorption of visible light can be increased. The photo-induced electrons can react with oxygen and water adsorbed on the surface to generate ˙O_2_^−^ and ˙OH, which make great contribution on degradation of MB.^43^ The porous structure and large specific surface area of CDAC can adsorb and enrich MB, and increase the concentration of MB in the composite, thus increasing the substrate concentration in the photocatalytic reaction region.^44^ Thus, the synergistic effect of effective charge separation, increased specific surface area, more light absorption and higher local MB concentration improves the photocatalytic activity of CDAC/W_18_O_49_ composites.

## 4. Conclusions

In conclusion, CDAC/W_18_O_49_ composites were synthesized by successfully assembling W_18_O_49_ nanowires onto CDAC prepared using KOH-active cow dung by co-hydrothermal treatment. The prepared CDAC/W_18_O_49_ composites showed higher photocatalytic activity and removal rate than pristine W_18_O_49_ nanowires in the full spectrum for MB. The large specific surface area and porous structure of CDAC in CDAC/W_18_O_49_ composites provide the W_18_O_49_ nanowires to absorb energy-generated excited electrons, which prolong the current load sub-life and accelerate the charge transfer; meanwhile, CDAC enhances the adsorption capacity of the composites to MB, thus improving the photocatalytic activity. Therefore, the prepared CDAC/W_18_O_49_ composite is an effective material for the photocatalytic degradation of methylene blue (MB).

## Conflicts of interest

There are no conflicts to declare.

## Supplementary Material
